# The Effect of Manufacturing Factors on the Material Properties and Adhesion of *C. albicans* and *S. mutans* on Additive Denture Base Material

**DOI:** 10.3390/ma18061323

**Published:** 2025-03-17

**Authors:** Laura Kurzendorfer-Brose, Martin Rosentritt

**Affiliations:** Department of Prosthetic Dentistry, UKR University Hospital Regensburg, 93042 Regensburg, Germany; martin.rosentritt@ukr.de

**Keywords:** printing, three-dimensional, dental materials, surface properties, *Candida albicans*, *Streptococcus mutans*

## Abstract

(1) Understanding the effects of manufacturing factors on microbial adhesion is essential for optimizing additive denture base materials and improving their clinical performance. This study evaluated how polymerization time, layer thickness, extended cleaning, and storage conditions influence *C. albicans* and *S. mutans* adhesion on a denture base material. (2) Specimens (n = 15/group, d = 8 mm, h = 2 mm) were additively fabricated or poured (reference). Digital light processing was performed with varying polymerization times, layer thicknesses, extended cleaning, and storage. Microbial adhesion was assessed using a luminescence assay. Surface properties were characterized by roughness (S_a_/S_z_), hardness, and surface free energy (SFE). Statistics: The Shapiro–Wilk test, ANOVA, Bonferroni post hoc test, and Pearson correlation (α = 0.05) were utilized. (3) Polymerization time, layer thickness, cleaning, and storage conditions significantly influenced *C. albicans* and *S. mutans* adhesion. Increased layer thickness reduced *C. albicans* adhesion but promoted *S. mutans* colonization, emphasizing the role of SFE. Extended polymerization and optimized cleaning reduced microbial adhesion, highlighting the need for tailored processing to enhance microbial resistance and material integrity. (4) Manufacturing factors influenced microbial adhesion, with additive materials reducing the abundance of *C. albicans* but increasing the abundance of *S. mutans*, underscoring the importance of material adjustments and extended polymerization to enhance microbial resistance.

## 1. Introduction

### 1.1. Candida albicans and Streptococcus mutans

The human oral cavity hosts a complex microbiome consisting of up to 700 species of microorganisms [1]. A variety of factors, including medication use and dietary habits, have been demonstrated to influence this microbiome, thereby contributing to the development of conditions such as dental caries. [2]. Among these microorganisms, the yeast *Candida albicans* (*C. albicans*) is a common commensal fungus that can become pathogenic under imbalanced oral conditions, leading to *Candida*-associated denture stomatitis [3,4,5,6,7], which affects 15–70% of denture wearers [5,8]. Poor denture hygiene and prolonged use of removable dentures facilitate *C. albicans* biofilm formation, increasing the risk of oral mucosal infections [3,4,5,9,10]. Similarly, the bacterium *Streptococcus mutans* (*S. mutans*) plays a key role in dental caries and periodontitis. *C. albicans* and *S. mutans* have been observed to coexist in cross-kingdom biofilms, a phenomenon that has been shown to enhance their growth and virulence, particularly in carious lesions [11]. This mutualistic interaction involves *S. mutans* supplying nutrients such as fructose and glucose to *C. albicans*, which in turn enhances carbohydrate metabolism in *S. mutans* [1,12]. This process increases acid production and accelerates root caries progression, elevating the risk of infection and caries development in partial denture wearers [11,13].

### 1.2. The Impact of Manufacturing Parameters on Material Properties

Advancements in computer-aided design and manufacturing (CAD/CAM) technologies have revolutionized dentistry, particularly in denture production [13]. Additive manufacturing, commonly known as 3D printing, offers a promising alternative to conventional processes such as cold polymerisation due to its time efficiency, cost-effectiveness, and adaptability [13,14,15]. In an additive printer, the materials can be present in different forms: liquid, powder, or solid. The state of the material depends on the used 3D printing technology. The digital light processing (DLP) technique is a widely used additive manufacturing method in dental medicine that utilizes liquid resin for fabrication [16]. In this process, the building platform is immersed in the liquid material and completely covered, and the prosthesis is produced upside down. A light source selectively cures the liquid resin at the designated object areas, polymerizing it layer by layer into a solid structure, where fabrication parameters influence material performance, such as the degree of polymerization [17,18,19]. Proper post-curing ensures complete polymerization and significantly impacts key material properties, including surface hardness and microbial resistance [20,21,22,23,24]. Research has demonstrated that extending the post-curing time can increase the hardness of additive denture base materials without altering surface roughness [25], making their properties comparable to those of poured materials.

However, *C. albicans* adhesion tends to be higher on additive materials, likely due to an increase in surface free energy (SFE) [10]. While uniform surface roughness does not appear to influence microbial colonization, reducing SFE and maintaining a layer thickness below 100 µm may help minimize *C. albicans* attachment [10,26]. Another study enhanced the mechanical and thermal properties of a polymethylmethacrylate (PMMA)-based denture base material by incorporating nanoparticles [27]. However, this study did not include microbial tests to assess the impact of nanoparticles on microbial adhesion. Another study demonstrated that the addition of nanoparticles effectively reduced *S. mutans* adhesion, while *C. albicans* adhesion increased [28]. However, this modification compromised the mechanical properties of heat-polymerized PMMA [28].

### 1.3. The Influence of Material Properties on C. albicans and S. mutans

The adhesion of *C. albicans* and *S. mutans* to denture surfaces is a significant factor in the development of infections [9]. Key surface properties, such as surface roughness and SFE, play a crucial role in biofilm formation [5,7,29], with higher surface roughness and SFE levels being associated with increased microbial adhesion and a greater risk of infections in denture wearers.

Recent research has increasingly focused on additive dental materials due to advancements in digital manufacturing technologies. Studies have investigated how polymerization processes, cleaning methods, and other fabrication parameters influence material properties, including surface roughness, SFE, and hardness [22,24,25,30,31]. However, the impact of these factors on microbial adhesion remains insufficiently explored, particularly concerning *C. albicans* and *S. mutans*.

While previous studies have examined the adhesion of *C. albicans* and/or *S. mutans* to poured, subtractive, and additive denture base materials, they often neglect the influence of specific additive manufacturing parameters, such as polymerization time, layer thickness, or storage conditions, on microbial resistance [10,31,32]. In general, additive materials frequently demonstrate discrepancies in mechanical properties and alterations following thermocycling and other ageing processes when compared to poured and subtractive materials [33,34,35].

### 1.4. Research Aim

A study has already shown that regular cleaning of dental prostheses is essential for effectively removing bacterial adhesion [36]. However, it is worth exploring whether manufacturing parameters can be adjusted during the additive manufacturing process of dental prostheses to both prevent microbial colonization and improve material properties. In this study, the most efficient production conditions for reducing microbial colonization are determined. Specifically, it investigates the effects of polymerization, layer thickness, cleaning, and storage conditions on the adhesion of *C. albicans* and *S. mutans* to denture base materials. The hypothesis is that these manufacturing adjustments, including polymerization time, layer thickness, cleaning, and storage conditions, influence surface properties and subsequently reduce microbial adhesion.

## 2. Materials and Methods

### 2.1. Specime Preparation

Round specimens (n = 15 per group; diameter: 8 mm; height: 2 mm) were fabricated using two different denture base manufacturing techniques to compare self-curing and additive manufacturing in terms of microbial adhesion and surface properties. The self-curing PMMA, processed via pouring, served as the reference material, while the DLP-printed additive material represented a modern alternative. The self-curing specimens were fabricated using the pouring technique (Palapress, Kulzer, Hanau, Germany), while the additive specimens (FotoDent denture 385 nm, pink/opaque, Dreve Dentamid, Unna, Germany) were produced using a DLP 3D printer (P30+ Straumann, Straumann, Freiburg, Germany) with a 90°printing orientation and a standard layer thickness of 50 µm. To examine the effects of additive manufacturing parameters on *C. albicans* and *S. mutans* adhesion, the following factors were varied: polymerization time, cleaning duration, and layer thickness. Additionally, the influence of storage conditions was examined (Table 1).

To ensure consistent and reproducible fabrication, all specimens underwent standardized polymerization and automated cleaning procedures. Normal cleaning (NC) and extended cleaning (EC) were performed with an automated cleaning unit (P wash, Straumann, Freiburg, Germany) and isopropanol to effectively remove unpolymerized resin. 

Polymerization was conducted using a light-curing unit (P cure, Straumann, Freiburg, Germany) with a fixed light intensity and exposure time, following the manufacturer’s recommendations. The specimens were randomly assigned to different polymerization conditions—short (SP), normal (NP), and long (LP)—to ensure uniform light exposure across all groups. 

After cleaning and polymerization, all specimens were polished with an automated precision grinding machine (Tegramin-25, Struers, Willich, Germany) under water cooling. A sequential grinding process was applied, starting with 1000-grade silicon carbide grinding paper (Strures, Willich, Germany), followed by 4000-grade (Buehler, Düsseldorf, Germany) to ensure a standardized surface treatment.

The post-processing storage conditions were controlled using a laboratory incubator (B6, Heraeus, Hanau, Germany), with specimens stored at controlled heat temperature (HT) for 7 days or in water for 30 days (W) (Table 2). All fabrication steps were conducted under identical environmental conditions to minimize variability and ensure reproducibility.

### 2.2. Surface Characterization

Surface roughness parameters, including the mean arithmetic height (S_a_) and maximum height (S_z_), were measured in accordance with ISO 25178-2:2019 [37] using a confocal 3D laser scanning microscope (CLSM; VK-100, Keyence, Osaka, Japan). The measurements were performed at 5× magnification over a square area of 2500 μm × 1900 μm, with cut-off wavelengths set to λ_s_ = 0.8 μm and λ_c_ = 0.08 mm. The measurement range was 7 mm, with a z-resolution of 0.005 μm, an x-resolution of 0.01 μm, and a repeatability of 0.02–0.05 μm.

Further testing was conducted using a universal hardness testing machine (ZwickiLine Z2.5, ZwickRoell, Ulm, Germany), following the recommendations of 6507-1:2024-01 [38]. The test was performed using a Vickers indenter, with a constant load rate of 0.1 mm/min and a maximum load of 20 N applied for 2 s. Hardness metrics, including Martens hardness (HM), indentation hardness (H_IT_), indentation modulus (E_IT_), and elastic indentation work (η_IT_), were calculated from force–indentation curves.

The surface free energy (SFE) was determined using a computer-assisted contact angle measuring device (DSA25, Krüss, Hamburg, Germany), in accordance with ISO 19403-2:2024 [39], which defines methods for measuring contact angles and SFE. The Owens and Wendt method [40] was applied, using the sessile drop technique with two liquids of varying hydrophobicity (Millipore water and diiodomethane, each with a drop volume of 1 µL). Before the measurements were taken, the specimens were cleaned with 70% ethanol to remove contaminants and ensure accurate SFE assessment.

To visualize the surface topography, a high-resolution image of each group (n = 1) was captured using a scanning electron microscope (SEM; Phenom, FEI Company, Eindhoven, Netherlands) at 1500× magnification, with a cut-off wavelength (λ_s_) of 439 nm and a filter wavelength (λ_c_) of 292.29 μm.

### 2.3. Adhesion

After surface characterization, the specimens were colonized with *C. albicans* and *S. mutans* to evaluate microbial adhesion [41]. *C. albicans* adhesion was quantified through luminescence measurements, while *S. mutans* adhesion was detected using fluorescence-based resazurin staining (Sigma-Aldrich, St. Louis, MO, USA). Prior to microbial inoculation, all specimens were cleaned with 70% ethanol and deionized water, followed by fixation in 48-well plates and the addition of 1 mL of sterile water.

For *C. albicans*, 1 mL of artificial saliva was applied to the specimens, followed by incubation for 2 h and 37 °C in a thermo-shaker (OrbitalShaker, Thermo Forma, Marietta, OH, USA). After removing the saliva, 1 mL of *C. albicans* suspension was added, and the specimens were incubated for another 2.5 h. Luminescence was then measured using a plate reader (Fluostar Optima, BMG Labtech, Offenburg, Germany).

For *S. mutans*, phosphate-buffered saline (PBS, 1 mL, Sigma-Aldrich, St. Louis, MO, USA) was removed after 24 h of incubation. The specimens were then exposed to artificial saliva for 2 h at 37 °C, after which 1 mL *S. mutans* suspension and 15 µL resazurin were added. After an additional 2.5 h incubation, the specimens were washed with PBS, and fluorescence intensity was measured with the plate reader.

### 2.4. Statistical Analysis

The Shapiro–Wilk test was used to asses data normality. The mean values and standard deviations were calculated. One-way ANOVA was performed to identify significant differences among groups, followed by the Bonferroni post hoc test. Pearson correlation analysis was used to evaluate relationships between microbial adhesion and surface parameters. All statistical analyses were performed using SPSS Statistics 29 (IBM, Armonk, NY, USA), with a significance level set at α = 0.05.

## 3. Results

The Shapiro–Wilk test indicated that approximately 84% of the data followed a normal distribution. ANOVA revealed statistically significant differences (*p* ≤ 0.049) between most groups, except for S_a_ and *C. albicans* adhesion. Significant results were observed for HM, H_IT_, E_IT_, η_IT_, SFE, and *S. mutans* adhesion (Bonferroni post hoc test, *p* ≤ 0.047).

### 3.1. Adhesion of C. albicans

**Figure 1:**
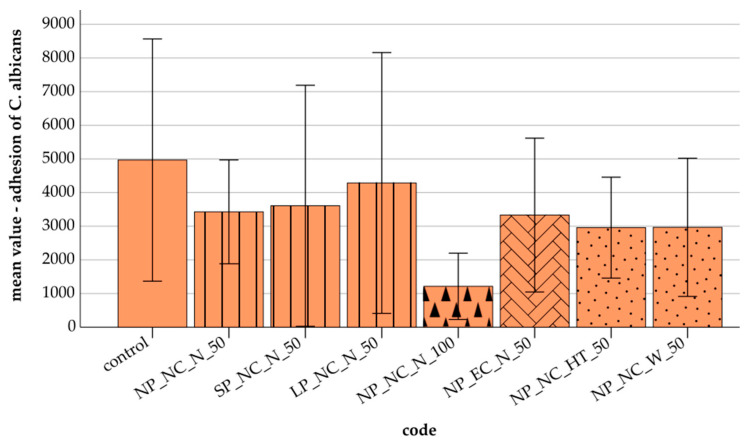
Mean value and standard deviations (error bars) of *C. albicans* adhesion under different conditions with 95% confidence intervals (CI): control (2391–7541); polymerization: **N**ormal **P**olymerization_NC_N_50 (2324–4531), **S**hort **P**olymerization_NC_N_50 (1046–6170), **L**ong **P**olymerization_NC_N_50 (1513–7058); layer thickness: NP_NC_N_**100** μm (509–1918); cleaning: NP_**E**xtended **C**leaning_N_50 (1696–4967); storage: NP_NC_**H**eat **T**reatment_50 (1885–4031), NP_NC_**W**ater_50 (1498–4436).

Figure 1 presents the adhesion results of *C. albicans* across the control and additively manufactured specimens. The control group exhibited the highest mean adhesion value (4966, SD = 3600). Extended polymerization time resulted in the highest adhesion as well as the largest standard deviation. Among the additively manufactured specimens, a layer thickness of 100 µm (NP_NC_N_100) showed the lowest *C. albicans* adhesion (1213, SD = 985). Cleaning had no discernible effect on adhesion, while storage tended to reduce adhesion levels. 

The Bonferroni post hoc test showed no statistically significant differences in the adhesion of *C. albicans* between the tested specimens (*p* = 1.000). However, a significant correlation was observed between *C. albicans* adhesion and S_a_ in NP_NC_N_100 (r ≤ 0.759, *p* ≤ 0.029). In NP_EC_N_50, *C. albicans* adhesion correlated significantly with HM (r ≤ 0.845, *p* ≤ 0.002), H_IT_ (r ≤ 0.830, *p* ≤ 0.003), and E_IT_ (r ≤ 0.782, *p* ≤ 0.008). 

### 3.2. Adhesion of S. mutans

**Figure 2:**
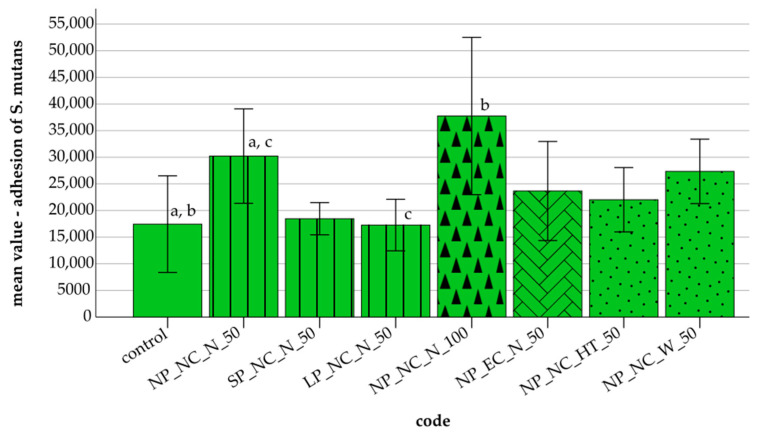
Mean value and standard deviations (error bars) of *S. mutans* adhesion under different conditions with 95% confidence intervals (CI): control (10,952–23,930); polymerization: **N**ormal **P**olymerization_NC_N_50 (23,879–36,559), **S**hort **P**olymerization_NC_N_50 (16,300–20,622), **L**ong **P**olymerization_NC_N_50 (13,805–20,730); layer thickness: NP_NC_N_**100** μm (27,179–48,303); cleaning: NP_**E**xtended **C**leaning_N_50 (17,010–30,315); storage: NP_NC_**H**eat **T**reatment_50 (17,702–26,341), NP_NC_**W**ater_50 (23,020–31,672). Identical letters represent significant differences within the control and additive groups (**a**,**b**) and between the additive groups (**c**) (*p* ≤ 0.05).

Figure 2 illustrates the adhesion of *S. mutans* across the tested specimens. Shorter and longer polymerization times resulted in reduced *S. mutans* adhesion compared to normal polymerization. Thicker layers (NP_NC_N_100) exhibited the highest adhesion and standard deviation (37,741, SD = 14,765). Extended cleaning led to a decrease in *S. mutans* adhesion, while storage generally reduced adhesion, with heat treatment in the incubator storage showing the most pronounced effect. 

Statistical analyses revealed significant differences in *S. mutans* adhesion among several groups (Bonferroni post hoc test, *p* ≤ 0.033). A significant correlation between *S. mutans* adhesion and SFE was found in the control group (r ≤ −0.761, *p* ≤ 0.028), NP_NC_N_50 (r ≤ 0.722, *p* ≤ 0.043), and LP_NC_N_50 (r ≤ 0.768, *p* ≤ 0.026). No significant correlations were observed between *C. albicans*/*S. mutans* adhesion and the other measured parameters in the remaining specimens.

### 3.3. Surface Parameters

The highest S_a_ value was observed in SP_NC_N_50 (3.48 µm, SD = 0.53), while the lowest was recorded in NP_EC_N_50 (2.98 µm, SD = 0.37), reflecting a difference of 0.59 µm. For S_z_, SP_NC_N_50 exhibited the highest value (27.48 µm, SD = 4.70), whereas LP_NC_N_50 had the lowest (22.23 µm, SD = 1.49), resulting in a variance of 5.25 µm (Table 3).

The hardness and elastic properties of the specimens are summarized in Table 4. The control group exhibited the highest mean values for HM (111.20 N/mm^2^, SD = 4.10), H_IT_ (145.10 N/mm^2^, SD = 5.72), and E_IT_ (3.97 kN/mm^2^, SD = 0.12). In contrast, NP_NC_W_50 showed the lowest mean values for HM (85.30 N/mm^2^, SD = 12.16), H_IT_ (118.80 N/mm^2^, SD = 15.22), and E_IT_ (2.47 kN/mm^2^, SD = 0.49). For η_IT_, NP_NC_W_50 recorded the highest mean value (39.16%, SD = 2.74), whereas the control group had the lowest (28.49%, SD = 0.32).

The highest SFE value was recorded in NP_NC_N_100 (36.28 mJ/m^2^, SD = 1.66), while the lowest was observed in NP_EC_N_50 (26.61 mJ/m^2^, SD = 2.29), resulting in a variation of 9.67 mJ/m^2^ (Table 5).

### 3.4. SEM Images

Surface depressions were noted across several specimens, with NP_NC_HT_50 exhibiting the most extensive surface depressions, while NP_EC_N_50 displayed the least prominent brightening in the images.

**Figure 3:**
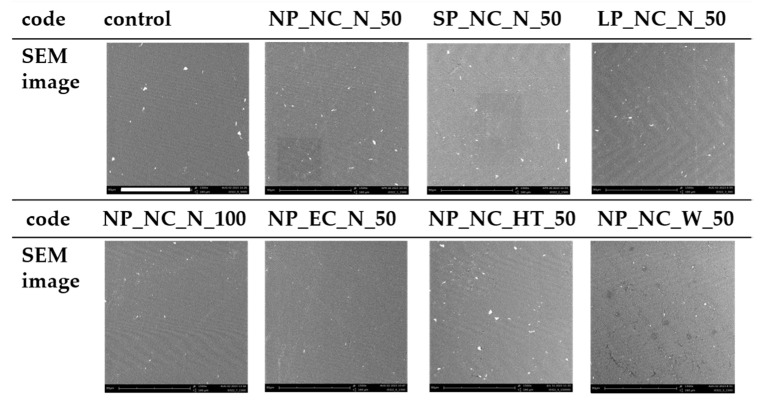
SEM images (magnification 1500×; scale bar (white): 80 µm refers to each figure) (polymerization: **N**ormal **P**olymerization_NC_N_50, **S**hort **P**olymerization_NC_N_50, **L**ong **P**olymerization_NC_N_50; layer thickness: NP_NC_N_**100** μm; cleaning: NP_**E**xtended **C**leaning_N_50; storage: NP_NC_**H**eat **T**reatment_50, NP_NC_**W**ater_50).

The SEM images revealed linear grinding marks on NP_NC_N_50, NP_NC_W_50, NP_EC_N_50, and the control, with NP_NC_N_50 exhibiting the most pronounced marks (Figure 3). These grinding patterns suggest variations in surface texture, which could impact microbial adhesion and material durability. Surface depressions were observed across several specimens, with NP_NC_HT_50 displaying the most extensive depressions, potentially indicating structural inconsistencies or increased porosity. In contrast, NP_EC_N_50 exhibited the least brightening in the images, suggesting a more uniform surface composition, which may influence its wettability and microbial colonization potential. These observations highlight the impact of different manufacturing factors and treatment conditions on surface morphology, which could have implications for microbial adhesion and long-term material performance.

## 4. Discussion

The hypothesis that manufacturing adjustments, including polymerization time, layer thickness, cleaning, and storage conditions, influence surface properties and subsequently reduce microbial adhesion was partially confirmed. This study demonstrates that modifying manufacturing parameters in additive materials had a significant effect on the adhesion behaviour of both *C. albicans* and *S. mutans*, although the influence on each of these microorganisms was not uniform.

### 4.1. C. albicans

Additively manufactured specimens generally exhibited lower *C. albicans* adhesion compared to the control group. Specimens subjected to normal polymerization demonstrated 1.4 times lower adhesion than the control. This finding aligns with the outcomes of an earlier study, which also reported a substantial reduction in *C. albicans* adhesion for additively manufactured materials compared to heat-polymerized specimens [42]. However, this study did not include comprehensive material characterization to fully substantiate these results. In contrast, previous studies have reported higher *C. albicans* adhesion on additively manufactured materials [32,43], potentially due to differences in the SFE of the materials [10]. The influence of surface roughness or hardness on *C. albicans* adhesion was observed only for specific fabrication parameters. 

#### 4.1.1. Polymerization

The polymerization time influenced *C. albicans* adhesion, with both shorter and longer polymerization durations leading to increased adhesion compared to normal polymerization. Interestingly, untreated specimens with a shorter polymerization time and lower surface roughness exhibited higher *C. albicans* adhesion than those subjected to prolonged polymerization with increased roughness [44]. This suggests that polymerization time has a greater impact on microbial adhesion than surface roughness. Contrary to previous research, the shorter polymerization time in this study increased surface hardness, a key factor for scratch resistance in dental materials. Since lower hardness can alter surface topography—particularly during mechanical aging—it may also contribute to bacterial adhesion [17]. Although extended polymerization is generally associated with increased hardness, and previous studies have reported that prolonged post-curing can achieve mechanical properties comparable to heat-polymerized materials [17,18,25], this could not be confirmed in the present study. Given that longer polymerization is expected to reduce residual monomer content, an increase in hardness would have been anticipated [17]. These findings underscore the importance of optimizing polymerization times to minimize fungal colonization on dental materials.

#### 4.1.2. Layer Thickness

Specimens with a 100 µm layer thickness exhibited the lowest *C. albiccans* adhesion and standard deviation, reducing fungal adhesion by a factor of 2.8 compared to the normal polymerization. Li et al. [26] recommended a layer thickness below 100 µm to minimize microbial adhesion, as their study found higher *C. albicans* adhesion and surface roughness at 100 µm, attributing this to pronounced grooves and ridges caused by the absence of surface treatment. In contrast, the surface treatment applied in this study effectively reduced roughness, which is likely to account for the observed differences in results. A statistically significant positive correlation was observed between *C. albicans* adhesion and S_a_ for specimens with a 100 µm layer thickness, confirming that increased roughness promotes fungal adhesion by providing a larger contact area for colonization. These findings suggest that increased layer thickness, combined with appropriate surface treatment, may be a strategic approach to reducing *C. albicans* colonization on denture base materials.

#### 4.1.3. Cleaning

A comparative analysis between normal polymerization and extended cleaning revealed negligible differences in *C. albicans* adhesion. A 5 min post-rinse has been shown to effectively remove cytotoxic monomers from additive materials without compromising material properties [45]. However, the prolonged cleaning time used in this study may not have been sufficient to have an effect on microbial adhesion. Prolonged cleaning (e.g., 12 h) carries a risk of surface damage and reduced material strength, making it unsuitable for clinical use [45]. Consequently, surface damage can lead to an increase in surface roughness, which, in turn, can result in enhancement of bacterial adhesion, thereby promoting the process of microbial colonization. Extended cleaning showed a significant positive correlation between *C. albicans* adhesion and hardness parameters (HM, H_IT_, and E_IT_), suggesting that harder and more elastic surfaces may facilitate *C. albicans* adhesion.

#### 4.1.4. Storage

*C. albicans* adhesion was slightly reduced by the storage conditions. Both incubator and water storage likely reduced residual monomer content, thereby mitigating cytotoxic effects and contributing to reduced microbial adhesion [46]. It was also observed that both storage conditions resulted in a reduction in surface roughness, which may have contributed to the observed decrease in adhesion, possibly due to a reduction in contact area. While water storage reduced *C. albicans* adhesion, prolonged immersion (30 d) resulted in material weakening, as evidenced by the lowest recorded hardness values. As emphasised by Rashid et al. [46], the effective removal of leachable monomers is optimised by short-term water storage (– d). The present findings are consistent with those of a previous study that reported an increase in adhesion and SFE after 50 h of water storage [10]. However, the discrepancy in the results could be explained by coarser surface treatments [10]. Long-term water storage highlights the degradation of material properties, emphasizing durability challenges in additive materials [22,30].

### 4.2. S. mutans

The control group exhibited lower *S. mutans* adhesion compared to the additively manufactured group, which contradicts previous results [42,47]. A significant negative correlation between *S. mutans* adhesion and SFE was observed in the control group, suggesting that materials with lower SFE may promote *S. mutans* adhesion. This trend was consistent with the findings for the additive specimen. The observed variations in this study may be attributable to disparities in the used materials, specifically the employment of heat-polymerized denture base material in prior studies, as contrasted with the utilization of cold-curing denture base material in this investigation. Additionally, differences in microbial test procedures may have influenced the adhesion outcomes.

#### 4.2.1. Polymerization

Adjustments to the polymerization process significantly influenced *S. mutans* adhesion. Normal polymerization resulted in the highest *S. mutans* adhesion, whereas both shorter and extended polymerization reduced adhesion, with the lowest levels observed for extended curing. This suggests that longer polymerization may create smoother, more compact surfaces, thereby inhibiting bacterial colonization. Aati et al. [17] found that longer polymerization reduces the number of residual monomers, which increases material stability and reduces cytotoxicity, which may also limit microbial adhesion. As reported by Poker et al. [47], the study authors found that surface roughness had no significant effect on the adhesion of *S. mutans*. Brambilla et al. [48] showed that extended polymerization reduces *S. mutans* colonization on composite resins, while Shim et al. [49] found that secondary curing significantly lowers residual monomers. This reduction may mitigate cytotoxic effects on microbial adhesion, improving material performance [46]. Similarly, Li et al. [24] have shown that the biocompatibility of 3D-printed resins varies depending on material composition and post-polymerization conditions. Their results highlight the importance of optimizing polymerization settings to achieve the lowest possible cytotoxicity and high antimicrobial efficacy. In contrast, materials subjected to prolonged polymerization (30 min) exhibited higher surface roughness and increased *S. mutans* adhesion on both untreated and polished specimens compared to those with a shorter polymerization time (6 min) [43]. 

Moreover, the results of another study indicated that the choice of post-polymerization technology has the capacity to influence the degree of cell toxicity associated with 3D-printed materials [50]. This is attributable to the release of elevated levels of residual monomers from materials that undergo insufficient curing processes. A significant positive correlation between *S. mutans* adhesion and SFE for both normal and prolonged polymerization suggests that lower SFE may inhibit *S. mutans* adhesion. The reduced adhesion observed with longer polymerization is likely due to a decrease in the number of residual monomers and lower surface roughness, further suppressing bacterial colonization.

#### 4.2.2. Layer Thickness

The layer thickness emerged as a critical factor, with the specimens exhibiting the highest levels of *S. mutans* adhesion among all tested groups. Despite the comparable levels of surface roughness and hardness observed at 50 µm and 100 µm layer thicknesses, specimens with a 50 µm layer thickness exhibited reduced adhesion and SFE. This finding underscores the predominant role of SFE in conjunction with layer thickness in promoting bacterial colonization. The higher SFE observed in thicker layers (100 µm) may have contributed to increased *S. mutans* adhesion. Additionally, two denture base materials with differing viscosities demonstrated contrasting results in surface roughness and contact angle measurements despite having untreated surfaces across both 50 µm and 100 µm layer thicknesses [51]. This suggests that material-specific properties interact with layer thickness, further influencing microbial adhesion.

#### 4.2.3. Cleaning

The cleaning treatment effectively reduced *S. mutans* adhesion. A longer cleaning process resulted in fewer surface contaminants and residual monomers, as evidenced by SEM images [45,52]. In addition, as used in this study, an automated purification method can more efficiently remove residual monomers and thus reduce cytotoxicity [50]. This removal may have smoothed surface irregularities, thereby reducing surface roughness. Additionally, extended cleaning could eliminate softer components, leading to an increase in surface hardness. However, prolonged cleaning may allow solvent molecules to penetrate the material, potentially altering its properties. After an extreme cleaning duration of 12 h, the formation of surface cracks was observed [45]. Consistently, longer cleaning durations have been reported to induce changes in material properties [45].

#### 4.2.4. Storage

Both heat treatment in an incubator and water storage were effective in reducing microbial adhesion; however, incubator storage was found to be more effective in this regard. Research has demonstrated that the process of water storage can effectively remove residual monomers from materials, which may consequently lead to a reduction in microbial adhesion [53,54,55]. This effect was also observed in this study, as evidenced by SEM images. However, it is possible that the material underwent a process of material aging as a consequence of prolonged water immersion, which resulted in a reduction in hardness. The absorption of water by the material could have increased SFE, further contributing to microbial adhesion reduction. Furthermore, it has been documented that elevated storage temperatures (e.g., 37 °C) reduced the residual monomer content, potentially enhancing microbial resistance [53]. Despite these effects, the application of controlled storage conditions has the potential to further optimize the antimicrobial properties of additive materials.

### 4.3. Limitation of the Study

The present in vitro study has inherent limitations as it does not fully replicate the dynamic oral environment, which consists of a diverse microbiota and mechanical forces. Furthermore, microbial adhesion was assessed over a relatively short period, limiting insights into long-term biofilm formation. Some unexpectedly high deviations limit the significance of the results. The investigation was restricted to a specific denture base material, which limits the transferability of the results to other additive materials. While surface roughness and SFE were evaluated, other influential parameters, such as chemical composition, may have been overlooked. Additionally, the limited range of manufacturing and cleaning parameters constrains a comprehensive understanding of their influence on microbial adhesion.

It is therefore recommended that future research efforts focus on expanding the range of investigated parameters to include variations in polymerization, material composition, and cleaning protocols. Furthermore, analyzing the residual monomer content under different manufacturing conditions and its impact on bacterial adhesion would provide valuable insights for optimizing microbial resistance in additive dental materials.

## 5. Conclusions

This study highlights the significant influence of manufacturing factors on the adhesion of *C. albicans* and *S. mutans* to denture base materials, demonstrating the complex interplay between microbial behavior and material properties. The findings reveal distinct adhesion patterns for the two microorganisms, which can be explained by specific interactions with SFE, roughness, and material composition. While additive manufacturing generally reduced *C. albicans* adhesion, it increased *S. mutans* adhesion, likely due to differences in SFE and topography. Higher SFE and smoother surfaces tend to promote *C. albicans* attachment, whereas *S. mutans* prefers microstructured surfaces that provide enhanced mechanical retention. These opposing trends emphasize the need for tailored material modifications to balance microbial resistance.

Extended polymerization, combined with controlled storage and cleaning protocols, can effectively reduce *C. albicans* adhesion by lowering residual monomer content and improving surface stability, potentially minimizing the risk of denture stomatitis in clinical applications. Similarly, prolonged polymerization also reduces *S. mutans* adhesion, likely due to increased crosslinking, which creates a denser polymer network and reduces surface irregularities, thereby limiting bacterial attachment.

These findings suggest that optimizing polymerization conditions and surface modifications can enhance the antimicrobial properties of denture base materials, reducing the risk of biofilm formation, plaque accumulation, and associated oral infections.

## Figures and Tables

**Table 1:** Abbreviations of the various influences.

Abbreviation	Influence
NP	Normal polymerization
SP	Short polymerization
LP	Long polymerization
NC	Normal cleaning (isopropanol)
EC	Extended cleaning (isopropanol)
50	Layer thickness of 50 µm
100	Layer thickness of 100 µm
N	No storage
HT	Heat treatment in an incubator at 37 °C (7 days)
W	Storage in water at 37 °C (30 days)

**Table 2:** Combination of the various influences.

Code	Material	Parameter
Control	Poured denture base material	Manufacturer’s recommendations
**Polymerization**		
**NP**_NC_N_50	Additive denture base material	NP-cure: Seq. 1: Wavelength: upper, 720 s, 80% Seq. 2: Wavelength: lower, 60 s, 100% NC: Pre-cleaning (4 min), cleaning (4 min), and drying (10 min) with isopropanol (99%)
**SP**_NC_N_50	Additive denture base material	SP-cure: Seq. 1: 360 s, 80% Seq. 2: 30 s, 100%
**LP**_NC_N_50	Additive denture base material	LP-Cure: Seq. 1: 1200 s (max. cure), 80% Seq. 2: 120 s, 100%
**Layer thickness**		
NP_NC_N_**100**	Additive denture base material	Layer thickness: 100 µm
**Cleaning**		
NP_**EC**_N_50	Additive denture base material	EC: Pre-cleaning (4 min), cleaning (4 min), and drying (10 min) with isopropanol (99%)
**Storage**		
NP_NC_**HT**_50	Additive denture base material	Heat treatment in an incubator: 7 d, 37 °C
NP_NC_**W**_50	Additive denture base material	Water storage in an incubator: 30 d, 37 °C

**Table 3:** Mean values and standard deviations (SDs) of surface roughness parameter S_a_ (µm) and S_z_ (µm) (polymerization: **N**ormal **P**olymerization_NC_N_50, **S**hort **P**olymerization_NC_N_50, **L**ong **P**olymerization_NC_N_50; layer thickness: NP_NC_N_**100** μm; cleaning: NP_**E**xtended **C**leaning_N_50; storage: NP_NC_**H**eat **T**reatment_50, NP_NC_**W**ater_50).

Code	S_a_ (µm)	S_z_ (µm)
Control	3.35 (0.54)	24.80 (3.26)
NP_NC_N_50	3.34 (0.48)	25.78 (3.78)
SP_NC_N_50	3.48 (0.53)	27.48 (4.70)
LP_NC_N_50	3.02 (0.14)	22.23 (1.49)
NP_NC_N_100	3.26 (0.52)	24.99 (3.87)
NP_EC_N_50	2.98 (0.37)	22.74 (2.55)
NP_NC_HT_50	3.01 (0.31)	22.70 (4.25)
NP_NC_W_50	3.13 (0.29)	24.11 (2.13)

**Table 4:** Mean values and standard deviations (SDs) of surface parameter HM (N/mm^2^), H_IT_ (N/mm^2^), E_IT_ (kN/mm^2^) and η_IT_ (%) (polymerization: **N**ormal **P**olymerization_NC_N_50, **S**hort **P**olymerization_NC_N_50, **L**ong **P**olymerization_NC_N_50; layer thickness: NP_NC_N_**100** μm; cleaning: NP_**E**xtended **C**leaning_N_50; storage: NP_NC_**H**eat **T**reatment_50, NP_NC_**W**ater_50).

Code	HM (N/mm^2^)	H_IT_ (N/mm^2^)	E_IT_ (kN/mm^2^)	η_IT_ (%)
control	111.20 (4.10)	145.10 (5.72)	3.97 (0.12)	28.49 (0.32)
NP_NC_N_50	96.60 (5.10)	131.00 (7.62)	3.06 (0.16)	35.92 (1.31)
SP_NC_N_50	99.00 (4.50)	133.10 (6.37)	3.19 (0.14)	34.34 (0.51)
LP_NC_N_50	90.40 (8.13)	122.90 (11.71)	2.89 (0.31)	36.43 (1.82)
NP_NC_N_100	97.00 (3.74)	130.70 (5.68)	3.14 (0.11)	35.05 (0.40)
NP_EC_N_50	98.30 (8.63)	132.10 (12.96)	3.20 (0.19)	35.07 (1.36)
NP_NC_HT_50	94.10 (8.72)	128.40 (12.07)	2.93 (0.37)	36.82 (1.97)
NP_NC_W_50	85.30 (12.16)	118.80 (15.22)	2.47 (0.49)	39.16 (2.74)

**Table 5:** Mean values and standard deviations (SDs) of surface parameter SFE (mJ/m^2^) (polymerization: **N**ormal **P**olymerization_NC_N_50, **S**hort **P**olymerization_NC_N_50, **L**ong **P**olymerization_NC_N_50; layer thickness: NP_NC_N_**100** μm; cleaning: NP_**E**xtended **C**leaning_N_50; storage: NP_NC_**H**eat **T**reatment_50, NP_NC_**W**ater_50).

Code	SFE (mJ/m^2^)
Control	34.16 (1.08)
NP_NC_N_50	30.66 (4.19)
SP_NC_N_50	35.44 (1.52)
LP_NC_N_50	31.91 (1.97)
NP_NC_N_100	36.28 (1.66)
NP_EC_N_50	26.61 (2.29)
NP_NC_HT_50	34.31 (4.40)
NP_NC_W_50	35.82 (1.98)

## Data Availability

The data presented in this article are available from the corresponding author upon request. The data are not publicly available due to privacy restrictions.
